# Alpha-Glucosidase and Alpha-Amylase Inhibitory Activities, Molecular Docking, and Antioxidant Capacities of *Plectranthus ecklonii* Constituents

**DOI:** 10.3390/antiox11020378

**Published:** 2022-02-14

**Authors:** Ninon G. E. R. Etsassala, Jelili A. Badmus, Jeanine L. Marnewick, Samuel Egieyeh, Emmanuel. I. Iwuoha, Felix Nchu, Ahmed A. Hussein

**Affiliations:** 1 Department of Horticultural Sciences, Cape Peninsula University of Technology, Symphony Road, Bellville, Cape Town 7535, South Africa; nchuf@cput.ac.za; 2 Applied Microbial and Health Biotechnology Institute, Cape Peninsula University of Technology, Symphony Road, Bellville 7535, South Africa; jabadmus@lautech.edu.ng (J.A.B.); marnewickj@cput.ac.za (J.L.M.); 3 School of Pharmacy, University of the Western Cape, Bellville 7535, South Africa; segieyeh@uwc.ac.za; 4 Chemistry Department, University of the Western Cape, Private Bag X17, Bellville 7535, South Africa; eiwuoha@uwc.ac.za; 5 Chemistry Department, Cape Peninsula University of Technology, Symphony Road, Bellville 7535, South Africa; mohammedam@cput.ac.za

**Keywords:** diabetes mellitus, alpha-glucosidase, alpha-amylase, oxidative stress, *Plectranthus ecklonii*

## Abstract

Shortage in insulin secretion or degradation of produced insulin is the principal characteristic of the metabolic disorder of diabetes mellitus (DM). However, because the current medications for the treatment of DM have many detrimental side effects, it is necessary to develop more effective antidiabetic drugs with minimal side effects. Alpha-glucosidase and alpha-amylase inhibitors are directly implicated in the delay of carbohydrate digestion. Pharmacologically, these inhibitors could be targeted for the reduction in glucose absorption rate and, subsequently, decreasing the postprandial rise in plasma glucose and the risk for long-term diabetes complications. The main objectives of this research study were to isolate different phytochemical constituents present in the methanolic extract of *Plectranthus* *ecklonii* and evaluate their alpha-glucosidase and alpha-amylase inhibitory activities and antioxidant capacity. The phytochemical investigation of the methanolic extract of *P. ecklonii* yielded three known compounds, viz. parvifloron D, F, and G (**1**–**3,** respectively). Parvifloron **G** was isolated for the first time from *P. ecklonii*. The in vitro bio-evaluation of the methanolic extract of *P. ecklonii* and its isolated compounds against alpha-glucosidase showed that **3** exhibited moderate inhibitory activity with IC_50_ values of 41.3 ± 1.2 μg/mL. Molecular docking analysis confirmed the alpha-glucosidase inhibitory activity demonstrated by **3**. Additionally, strong antioxidant capacities were demonstrated by **3** and **1** on ORAC (28726.1 ± 8.1; 3942.9.6.6 ± 0.1 µM TE/g), respectively, which were comparable with the reference antioxidant epigallocatechingallate (EGCG). Furthermore, **3** also showed strong activity on TEAC (3526.1 ± 0.6 µM TE/g), followed by **2** (1069.3 ± 2.4 µM TE/g), as well as on FRAP (1455.4 ± 2.0 µM AAE/g). The methanolic extract of *P. ecklonii* is a rich source of abietane diterpenes with strong antioxidant activities. This is the first scientific report on alpha-glucosidase and alpha-amylase inhibitory activities, molecular docking, and antioxidant capacities of *P. ecklonii* constituents.

## 1. Introduction

Diabetes mellitus (DM) is a group of systemic metabolic disorders with a high rate of morbidity and mortality worldwide [1,2]. Hyperglycemia is the main consequence of DM, which results from a deficiency in insulin secretion or degradation of produced insulin [3]. The chronic hyperglycemia of diabetes may result in long-term damage, dysfunction, and failure of several organs, such as eyes, kidneys, nerves, heart, and blood vessels. Many factors (internal and external), such as obesity, sedentary lifestyle, and oxidative stress, are directly involved in these cell alterations [4,5]. However, the occurrence of oxidative stress happens when the production of free radicals overwhelms the detoxification capacity of the cellular antioxidant system, resulting in biological damage such as microvascular and cardiovascular complications [6]. Regrettably, there is currently no cure available for diabetes, but it can be managed by controlling blood sugar levels through a healthy diet, exercise, and medication, which can reduce the risk of long-term diabetes complications [7]. Therefore, there is a great need in developing alternative natural antidiabetic products with a high safety margin. Medicinal plants have been utilized as sources of medicine since ancient times [8], and a number of them have been used in the treatment of diabetes, including *Plectranthus species* such as *P. amboinicus, P.* madagascariensis, and *P. amboinicus* [9,10,11].

*P. ecklonii* is a shrub that grows up to 3 m, and it is widely distributed in South Africa, Australia, New Zealand, Mexico, and the United States. In South Africa, *P. ecklonii* has a wide distribution ranging from Somerset east in the Eastern Cape to Barberton in Mpumalanga. The leaves of this plant are spread out in opposite pairs on the square stems, the hindmost with tufts of purplish hairs along the nodes. *P. ecklonii* grows predominately in semi-shade areas, understory at forest margins, or on wooded stream banks during rainfall season, especially summer. It is traditionally used in South Africa to treat different kinds of diseases, such as stomachaches, nausea, vomiting, and meningitis [12]. The aerial part (leaves) of the plants is also utilized for respiratory abnormalities, chest problems, and coughs (TB-related problems) [13]. Aerial parts of the plant are used in Zimbabwe for the treatment of skin diseases and skin hyper-pigmentation problems [14]. 

Numerous compounds have been isolated from the leaves of *P. ecklonii,* including parvifloron D and F [14], rosmarinic acid [15], vitexin, isovitexin, apigenin 7-*O*-β-glucoside, apigenin 4′,6-dimethoxy-7-*O*-β-glucoside, luteolin 7-*O*-β-glucoside, apigenin, luteolin, and caffeic acid [16]. 

Parvifloron D (**1**) has been reported to show selective cytotoxicity to pancreatic cell lines and can be used as a possible efficient alternative approach in the treatment of pancreatic cancer [17]. It also showed strong antimicrobial activity and cytotoxicity [18]. Furthermore, parvifloron D is a potent cytotoxic compound against several human tumor cells and a fast and strong apoptotic inducer in leukemia cells [12]. Parvifloron F (**2**) showed antiproliferative activity against triple-negative breast cancer [19] and antibacterial activity [14]. *P. ecklonii* has also been reported to possess a wide range of pharmaceutical applications, including antimicrobial, antioxidant, antiacetylcholinesterase, antifungal, and anti-inflammatory activities [15].

This research work primarily examined the phytochemical isolation of different constituents present in the methanolic extract of *P. ecklonii,* as well as being an in vitro bio-evaluation of the total extract and its isolated compounds against alpha-glucosidase and alpha-amylase enzymes, in addition to examining their antioxidant properties.

## 2. Materials and Methods

### 2.1. Materials

Trolox (6-hydroxyl-2,5,7,8-tetramethylchroman-2-carboxylicacid), EGCG (epigallocatechin gallate), and other reagents such as ABTS (2,2-azino-bis (3-ethylbenzo thiazoline-6-sulfonic acid) diammonium salt), TPTZ (2,4,6-tri[2 -pyridyl]-s-triazine), iron (III) chloride hexahydrate, AAPH (2,2-Azobis(2-methylpropionamidine) dihydrochloride), fluorescein sodium salt, potassium peroxodisulfate, copper sulfate, hydrogen peroxide, perchloric acid, alpha-glucosidase (Saccharomyces cerevisiae), alpha-amylase (procaine pancreas), and 3,5,di-nitro salicylic acid (DNS), sodium carbonate (Na_2_CO_3_), 4-nitro-phenyl-α-D-glucopyranoside (pNPG), di-sodium hydrogen phosphate, and sodium dihydrogen phosphate were purchased from Sigma-Aldrich, Cape Town, South Africa. The organic solvents used in this study were supplied by Merck, Cape Town, South Africa. The NMR spectra were carried out on an Avance 400 MHz spectrometer (Bruker, Rheinstetten, Germany) using deuterated chloroform. Preparative HPLC was utilized for the purification of compounds using HPLC-grade methanol and distilled water.

### 2.2. Plant Material

The aerial part of *P. ecklonii* (leaves) was collected in February 2019, from the Cape Peninsula University of Technology campus, Bellville, South Africa. A voucher specimen was identified at the Compton Herbarium, Kirstenbosch, by Prof. Christopher Cupido, with the herbarium number NBG1465544-5.

### 2.3. Extraction and Purification of Different Chemical Constituents from P. ecklonii

The aerial part of the fresh plant material (932.3 g) was blended and extracted with methanol (2.5 L) at room temperature (approx. 25 °C) for 24 h. The methanol extract was filtered and evaporated to dryness under reduced pressure at 40 °C to yield 61.2 g (6.56 %). The total extract (60 g) was subjected to silica gel column chromatography and eluted using the gradient of Hex and EtOAc in order of increasing polarity. Fifty-one (51) fractions (250 mL each) were collected during the process and numbered (1–51). The collected fractions (1–51) were concentrated and combined according to their TLC profiles using Hex/EtOAc (7:3) and Hex/EtOAc (8:2) to yield eight (8) main fractions coding with roman numbers (I–VIII). Main fraction III (3615 mg) was subjected to successive silica gel columns using Hex/EtOAc gradient (9:1; 7:3; and 100%). Fractions of 50 mL each were collected and evaporated using a rotary evaporator. Fractions obtained were developed on TLC, and the fractions that displayed the same profiles were combined.

Fractions 41–43 and 44–47 were suspected to be pure due to having a single spot after development on the TLC plate. These fractions were pooled together, evaporated, and re-spotted to further confirm the purity of the combined fractions. The TLC showed only a single spot after development, confirmed the purity, and labelled the compounds as (**1**, 415.8 mg, 0.045%) and (**2**, 322.7 mg, 0.035%).

Sub-fraction PE-III-2 (54.5 mg) was injected into the HPLC and eluted using a gradient of MeOH and de-ionized water (50:50 to 100% MeOH in 60 min), which afforded a prominent peak that was collected and labeled as PE-III-2-1. After spotting and developing the fractions on the TLC plate, a single spot suspected to be a pure compound was observed. The fractions that afforded this single spot were pooled together and labeled as (**3,** R_t_ 42.1 min, 29.9 mg, 0.032%).

### 2.4. Molecular Docking of Isolated Compounds with Alpha-Glucosidase

Molecular docking was used to study the in silico molecular interaction of the protein target with the ligands. The protein target was the three-dimensional model of alpha-glucosidase, while the ligands were the isolated compounds. The crystal structure of the protein target was downloaded from the RSCB protein data bank (PDB), code number 5NN5. The protein preparation module in Molecular Operating Environment MOE 2019.01 was used to prepare the three-dimensional structure of alpha-glucosidase (5NN5) for virtual screening. The binding site was defined as the region occupied by the co-crystallized ligand in the three-dimensional structure of alpha-glucosidase. The ligands were also prepared for virtual screening by washing, protonation, and minimization of the chemical structures using Molecular Operating Environment MOE 2019.01. The chemical structure of acarbose, a known inhibitor of alpha-glucosidase, was included for virtual screening as a control.

Molecular docking simulation was carried out with the MOE Dock module using the induce-fit model. The three-dimensional structure of alpha-glucosidase was set as ‘Receptor’. The ‘Triangle Matcher’, which is suitable for standard and well-defined binding sites, was set as the ligand placement method. Thirty poses were generated for each ligand, and the poses were scored according to the London ΔG scoring function. The thirty poses were taken through molecular mechanics (MM) refinement to get ten final poses. The final docking score was evaluated with the GBVI/WSA ΔG scoring function with the Generalized Born solvation model (GBVI) (Onufriev, A.V.; Case, D.A. Generalized Born implicit solvent models for biomolecules. Annual review of biophysics. **2019**, *48*, 275–96. pmid:30857399). MOE and UCSF ChimeraX [20]. (UCSF ChimeraX: Structure visualization for researchers, educators, and developers were used to visualize the poses and ligand interaction with the protein.

### 2.5. Physicochemical and Pharmacokinetic Characterization of Isolated Chemical Constituents

The in-silico prediction of the physicochemical and pharmacokinetic properties of the isolated compounds was performed using the online software: SwissADME (https://swissadme.ch 16 August 2021) [21]. The physicochemical parameters (lipophilicity (logP), molecular weight, polar surface area, number of hydrogen bond donors and acceptors, number of rotary bonds, and solubility in water), drug-likeness profile, pharmacokinetic profile (absorption, distribution, metabolism, excretion, and toxicity) were obtained for the three isolated compounds and acarbose.

### 2.6. Biological Characterization

#### 2.6.1. Alpha-Glucosidase Inhibitory Activity

The alpha-glucosidase inhibitory activity of the isolated compounds (**1**–**3**) was carried out according to the standard method with slight modification [22]. In a 96-well plate, the reaction mixture containing 50 μL of phosphate buffer (100 mM, pH 6. 8), 10 μL of alpha-glucosidase enzyme (1 U/mL), and 20 μL of varying concentrations (133.33–4.17 μg/mL) of isolated compounds were pre-incubated at 37 °C for 15 min. Then, 20 μL of PNPG (5 mM) was added as a substrate and incubated at 37 °C for 20 min. The reaction was stopped by adding 50 μL of sodium carbonate Na_2_CO_3_ (0.1 M). The absorbance of the released p-nitrophenol was measured at 405 nm using a Multiplate Reader (Multiskan Thermo Scientific, version 1.00.40, Vantaa, Finland). Acarbose at various concentrations was included as a standard. Each experiment was repeated three times. The results are expressed as percentage inhibition, which was calculated using the formula

Inhibitory activity (%) = (1 − A/B) × 100

where A is the absorbance in the presence of test substance, and B is the absorbance of control.

#### 2.6.2. Alpha-Amylase Inhibitory Activity

The alpha-amylase inhibitory activity of the isolated compounds (**1**–**3**) was carried out according to the standard method with slight modification [22]. In a 96-well plate, the reaction mixture containing 50 μL of phosphate buffer (100 mM, pH 6.8), 10 μL of alpha-amylase enzyme (2 U/mL), and 20 μL of varying concentrations (100–31.2 μg/mL) of isolated compounds was pre-incubated at 37 °C for 20 min. Then, 20 μL of 1% soluble starch (100 mM phosphate buffer pH 6.8) was added as a substrate and incubated at 37 °C for 30 min. A volume of 100 μL of the color reagent (DNS) was added and then boiled for 10 min. The absorbance of the resulting mixture was measured at 540 nm using a Multiplate Reader (Multiskan Thermo Scientific, version 1.00.40, Vantaa, Finland). Acarbose at various concentrations was used as a standard. Each experiment was repeated three times. The results are expressed as percentage inhibition, which was calculated using the formula

Inhibitory activity (%) = (1 − A/B) × 100

where, A is the absorbance in the presence of test substance, and B is the absorbance of control.

### 2.7. Antioxidant Assays

#### 2.7.1. Ferric-Ion Reducing Antioxidant Power (FRAP) Assay

The FRAP assay was carried out in accordance with the method described previously [23]. In a 96-well plate, 10 μL of the stock solution (1 mg/mL *w*/*v*) of the isolated compounds (**1**–**3**) was mixed with 300 μL of FRAP reagent. The FRAP reagent was prepared by mixing (10:1:1, *v/v/v*) of acetate buffer (300 mM, pH 3.6), tripyridyl triazine (TPTZ) (10 mM in 40 mM HCl), and FeCl_3._6H_2_O (20 mM). Incubation commenced at room temperature for 30 min, and the plate was read at a wavelength of 593 nm in a Multiskan spectrum plate reader (Thermo Fisher Scientific). L-Ascorbic (Sigma-Aldrich, Cape Town, South Africa) was used as a standard, with concentrations varying between 0 and 1000 μM. Further dilutions were carried out with the samples that were highly concentrated, and such dilution factors were recorded and used for calculations of the affected samples. The results are expressed as μM ascorbic acid equivalents per milligram dry weight (μM AAE/g) of the test samples.

#### 2.7.2. Automated Oxygen Radical Absorbance Capacity (ORAC) Assay

ORAC assay was conducted according to the previous method with slight modifications [24]. The method measured the antioxidant scavenging capacity of thermal decomposition generated by peroxyl radical of 2,2-azobis (2-amino-propane) dihydrochloride (AAPH) as peroxyradical (ORAC_ROO_^.^) generator. The loss of fluorescence of fluorescein (probe) was an indication of the extent of its oxidation through reaction with the peroxyl radical. The protective effect of an antioxidant was measured by assessing the fluorescence area under the curve (AUC) plot relative to that of blank in which no antioxidant was present. The analyzer was programmed to record the fluorescence of fluorescein every 2 min after AAPH was added. The fluorescein solution and sample were added in the wells of an illuminated 96-well plate, and 12 μL of each sample in a stock solution of 1 mg/mL was combined with 138 μL of a fluorescein working solution, followed by the addition of 50 μL of 150 mg of AAPH prepared in situ in 6 mL phosphate buffer. Absorbance was measured with a Fluoroskan spectrum plate reader with the excitation wavelength set as 485 nm and the emission wavelength at 530 nm. A calibration curve was used, using a Trolox stock solution of concentration in the range of 83–417 μM (R^2^ = 0.9514). The ORAC values were calculated using a regression equation (Y = a + bX + Cx^2^) between Trolox concentration (Y in μM) and the net area under the fluorescence decay curve (X). ORAC values are expressed as μMTE/mg of a test sample. Samples without perfect curve were further diluted, and the dilution factors were used for the calculation of such samples.

#### 2.7.3. Trolox Equivalent Absorbance Capacity (TEAC) Assay

The TEAC assay was carried out in accordance with the method described previously [25]. The stock solutions, which contained 7 mM of ABTS and 140 mM of K_2_S_2_O_8_, were prepared and kept at −2 °C. The working solution was then prepared by adding 88 μL of K_2_S_2_O_8_ solution to 5 mL of ABTS solution. The two solutions were mixed and allowed to react for 24 h at room temperature in the dark. Trolox was used as a standard, with concentrations ranging between 0 and 500 μM. After 24 h, the ABTS mix solution was diluted with ethanol to read a start-up absorbance (control) of approximately 2.0 (±0.1). The stock solution (1 mg/mL) of a methanol extract and purified compounds (25 μL) were allowed to react with 300 μL of ABTS in the dark at room temperature for 30 min. The absorbance was read at 734 nm at 25 °C in the plate reader. The results are expressed as μM Trolox equivalents per milligram dry weight (μM/TE/g) of the test samples.

### 2.8. Statistical Analysis

All the measurements were repeated three times, and the IC_50_ values were calculated using GraphPad Prism 6 version 6.01 (Graph pad software, Inc., La Jolla, CA, USA.) statistical software. The data presented are means ± SD obtained from three independent in vitro experiments on 96-well plate readers. Data were analyzed using one-way ANOVA, followed by Tukey’s multiple comparisons test.

## 3. Results and Discussion

### 3.1. Chemical Characterization

**Figure 1:**
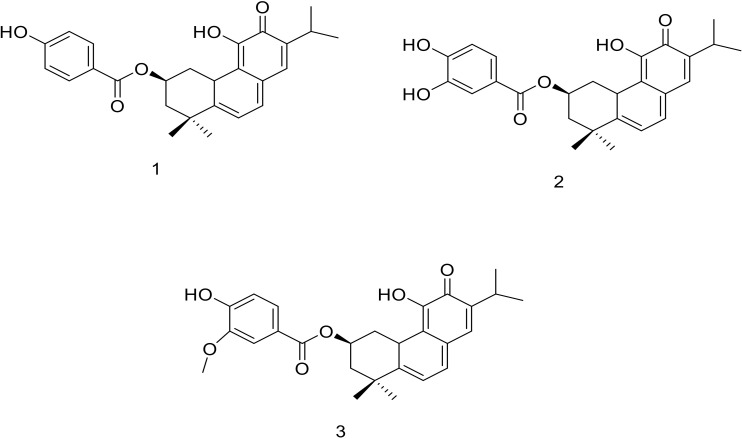
Chemical structures of the isolated compounds.

Repeated silica gel and Sephadex column chromatography of the methanolic extract of *P. ecklonii* yielded three known terpenoids (Figure 1). The structures of compounds (**1**–**3**) were confirmed by comparison of the spectroscopic data with literature data.

#### 3.1.1. Structure Elucidation of Parvifloron D (**1**)

Compound **1** was isolated as orange powder. It was identified as parvifloron D based on its NMR data, which showed typical abietane diterpene signals. The ^13^C NMR and DEPT spectra confirmed the presence of twenty-seven (27) carbons consisting of 20 carbons belonging to the diterpene skeleton and 6 of which belong to the signals of p-benzoic acid derivatives. The signal of 2β was shifted to low field due to the esterification of the p-benzoic acid with the OH at the same position. The ^1^H NMR signals showed five methyl groups signals at 1.16, 1.18 (*d/*each, *J* = 2.8, Me-16,17), 1.30 (*s*, Me-18), 1.43 (*s*, Me-19), and 1.66 (*s*, Me–20), in addition to signals at 6.42 (*d*, *J* = 6.8; H-6), 6.79 (*d*, *J* = 6.8, H-7), 6.89 (*d*, *J* = 8.6, H-14), and 6.97 (*s*, H-2β). The identity of the compound was confirmed by comparing the experimental data with literature [17]. This compound was previously isolated from *P. strigosus* Benth [14].

#### 3.1.2. Structure Elucidation of Parvifloron F (**2**)

Compound **2** was isolated as yellow powder. It was identified as parvifloron F based on its NMR data, which showed close similarity with **1** except for the side chain, which exhibited signals of 1,3,4-trisubstituted benzoic acid patterns instead of 1,4-disubstituted benzoic acid. The identity of the compound was confirmed by comparing the experimental data with literature [26]. This compound was previously isolated from *P. nummularius* Briq [14].

#### 3.1.3. Structure Elucidation of Parvifloron G (**3**)

Compound **3** was isolated as yellow brownish powder. It was identified as parvifloron G based on its NMR data, which showed close similarity with **1** and **2** except the presence of the methoxyl group at 3.89 ppm on the benzoic acid. The identity of the compound was confirmed by comparing the experimental data with literature [27]. This is the first report on the isolation of **3** from *P. ecklonii.* This compound was previously isolated from *P. strigosus* [27].

### 3.2. Biological Evaluation

#### 3.2.1. Alpha-Glucosidase and Alpha-Amylase Activities

The search for antidiabetic compounds from natural sources has been intensified and a great deal of research is being conducted to identify plants/compounds with prominent antidiabetic activity, with emphasis on the inhibition of the two enzymes: alpha-glucosidase and alpha-amylase. In this study, the inhibitory activity of isolated compounds from *P. ecklonii* was investigated, and the results show that **3** is the only compound that exhibited significant (*p* < 0.0001) alpha-glucosidase inhibitory activity, with an IC_50_ value of 41.3 ± 1.2 µg/mL. The crude extract of *P. ecklonii* showed some activity against alpha-glucosidase enzyme with an IC_50_ value of 145.4 ± 3.1 µg/mL, as shown in Table 1. Compounds **1**, **2**, and **3** shared the same chemical backbone except the presence of the methoxyl group on the benzoic acid in **3**, which might be responsible for the moderate bioactivity demonstrated.

#### 3.2.2. Antioxidant Activity

Oxidative stress is mainly caused by an over-accumulation of free radicals, which are produced by the body via various endogenous systems, such as exposure to various physiochemical conditions or pathological states. Hence, free radicals can alter lipids, proteins, and DNA and can set off a number of human diseases, including diabetes [28]. One of the therapeutic approaches to combat these diseases is to search for potential antioxidant candidates that can assist in managing oxidative stress and other related diseases [29]. The in vitro bio-evaluation of the antioxidant activity of the isolated compounds of the methanolic extract of *P. ecklonii* was investigated by measuring their ORAC, TEAC, and FRAP activities. The results demonstrate that **3** and **1** exhibited strong antioxidant activity on ORAC (28,726.1 ± 8.1; 3942.9 ± 7.1) µM TE/g, respectively, when compared with the reference antioxidant, positive control (EGCG). Compounds **3** also showed strong activity on TEAC (3526.1 ± 0.6) µM TE/g, followed by **2** (1069.3 ± 2.4) µM TE/g, as well as on FRAP (1455.4 ± 2.0) µM AAE/g, as shown in Table 2. The antioxidant activity of **3,** considering ORAC, TEAC, and FRAP results, was significantly (*p* < 0.0001) higher when compared with **1** and **2**. This implies that the additional methoxyl group of **3** confered the observed antioxidant activity. Additionally, the crude extract of *P. ecklonii* exhibited excellent free radicals scavenging effect with an IC_50_ value of 54.8 ± 1 µg/mL, which justifies the bioactivity demonstrated by its chemical constituents.

Parvifloron D (**1**) has been reported to have strong antioxidant activity when evaluated for its ability to scavenge the DPPH radical (IC_50_ = 1.3 ± 0.1 mM), and its structure-activity relationship might be ascribed to fact that it can generate a molecule with greater resonance stabilization that can easily scavenge the DPPH radical [28]. It has been reported that **2** scavenges the DPPH radical more than tocopherol (positive control) with an EC_50_ value of 0.131 mM [26].

### 3.3. Molecular Docking of Isolated Compounds and Acarbose with Alpha-Glucosidase

**Figure 2:**
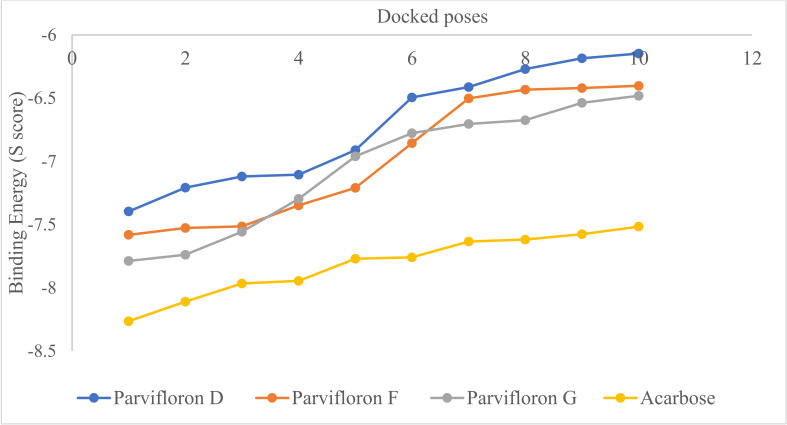
Top ten docked poses of parvifloron D, parvifloron F, parvifloron G, and acarbose on the x-axis plotted against the binding affinity (S score) for alpha-glucosidase. The greater the negative value of S score, the stronger the predicted binding affinity for alpha-glucosidase. Among the isolated compounds, parvifloron G showed more poses with lower S scores.

The molecular docking study on isolated compounds and acarbose (a reference) revealed that all the ten poses of acarbose showed lower S score than the isolated compounds (Figure 2), suggesting that acarbose will have the strongest binding affinity to the enzyme; alpha-glucosidase thereby might show the greatest inhibition of the enzyme. Amongst the three isolated compounds, the top ten docked poses of parvifloron F and parvifloron G showed slightly lower S score than the top ten docked poses of parvifloron D. This suggests that parvifloron F and parvifloron G (particularly parvifloron G, which showed more poses with lower S score) might have a slightly greater inhibition of alpha-glucosidase.

**Figure 3:**
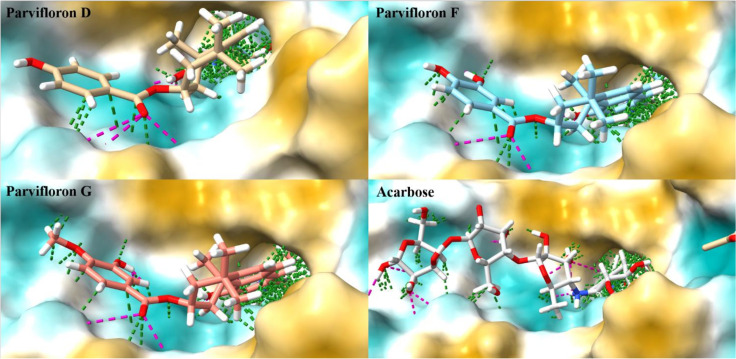
A detailed look into the binding of the top docked poses of parvifloron D, parvifloron F, parvifloron G, and acarbose to the macromolecular target, alpha-glucosidase, revealed hydrogen bonds (purple dotted lines) and Van der Waals interactions (green dotted lines) with amino acid residues at the binding site. The molecular lipophilicity potential (MLP) for the binding site of alpha-glucosidase is shown. The coloring on the molecular surface ranges from dark cyan (most hydrophilic) to white to dark goldenrod (most lipophilic).

**Figure 4:**
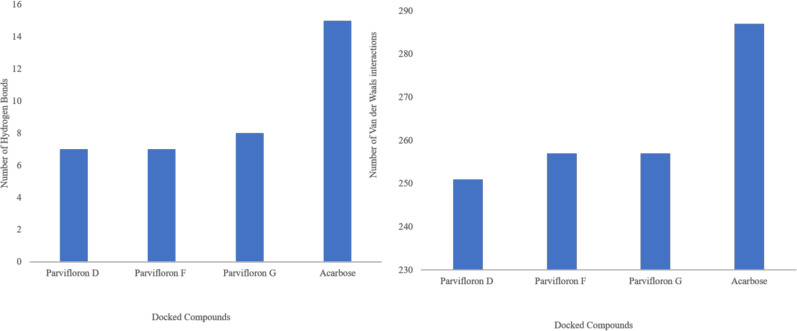
A count of the various types of interactions (hydrogen bonds and Van der Waals) of the isolated compounds and acarbose with the binding site of alpha-glucosidase.

In a detailed look into the binding of the top docked poses of the isolated compounds and acarbose to the macromolecular target, alpha-glucosidase revealed hydrogen bond (at a minimum distance of 2.5 Å and 20° angle) and Van der Waals (at minimum overlap of −0.4 Å) interactions with amino acid residues at the binding site (Figure 3). A count of the various types of interactions with the binding site of alpha-glucosidase (Figure 4) showed that the reference compound acarbose had the most number and types of interactions. Parvifloron D and parvifloron F showed a total of seven hydrogen bond interactions while parvifloron G showed eight hydrogen bond interactions (Figure 2). These results suggest that although the reference compound acarbose might have the strongest interaction with alpha-glucosidase, parvifloron G showed the highest affinity for the target—higher than parvifloron F and parvifloron D.

Overall, the molecular docking studies provide insight into the potential of the isolated compounds to inhibit alpha-glucosidase and, also provide insight into potential strategies for structure-based design or optimization of these isolated compounds or new compounds with better inhibitory activity against alpha-glucosidase.

### 3.4. Physicochemical and Pharmacokinetic Characterization of Isolated Compounds

One of the main limitations to progress along the drug development pipeline is poor physicochemical and pharmacokinetic properties. Low or no toxicity, good oral bioavailability, and optimum physicochemical properties are key parameters that will reduce the chance for attrition of compounds during drug development for any disease. In the present work, the three isolated compounds from *P. ecklonii* and acarbose were evaluated for their pharmacokinetic properties. The results (Table 3) show that acarbose had higher values than the three isolated compounds for key physicochemical properties, such as molecular weight, number hydrogen bond donors, number of hydrogen bond acceptors, number of rotatable bonds, and total polar surface area. These properties are important features in various drug-likeness and bioavailability models (e.g., Lipinski rule of five, Veber model, and Egan model) that determine the drug-likeness and pharmacokinetic properties of compounds. For example, the logP was significantly lower for acarbose compared with the three isolated compounds, suggesting that acarbose is more hydrophilic, while these compounds are lipophilic. As a result, acarbose was predicted to have low gastrointestinal (GI) absorption, while the isolated compounds were predicted to have high GI absorption.

Overall, acarbose showed the highest values, above the ideal values for the drug-likeness or good bioavailability, for most of the physicochemical properties predicted, suggesting that it will not be readily bioavailable. This is consistent with current observation and the use of acarbose as an alpha-glucosidase inhibitor within the gastrointestinal tract. Conversely, the values of the predicted physicochemical properties for the isolated compounds were all within the ideal values for drug-likeness or good bioavailability suggesting that they will be readily bioavailable (as seen with the GI absorption and bioavailability score prediction).

A look at the potential to inhibit the cytochrome P450 (CYP450) isoenzymes revealed that the isolated compounds were predicted to inhibit only CYP2C9. The potential to inhibit CYP2C9 enzyme suggests that these compounds may limit the metabolism of some current drugs (including anticoagulants such as warfarin, oral antidiabetic drugs such as glipizide, antiepileptics such as phenytoin, and nonsteroidal anti-inflammatory drugs such as ibuprofen) when co-administered and might lead to toxicity due to increased plasma concentration of such drugs. Overall, the isolated compounds showed physicochemical and pharmacokinetic properties that were within the ideal limit of drug-likeness.

PAINS (pan assay interference compounds) alerts are substructures that are likely to interfere in bioactivity screening via several means but particularly through protein reactivity. One PAINS alert (catechol A) was predicted to be present in the isolated compounds, while acarbose had none. PAINS alerts represent poor choices for drug development, though some authors have proposed that they may be suggestive of a selective and optimizable hit.

Finally, a toxicity risk assessment that tried to locate substructures within the chemical structure that were indicative of a toxicity risk found a high risk of toxicity for the three isolated compounds but no risk for acarbose. Although risk alerts are by no means meant to be a fully reliable toxicity prediction, particular attention and toxicity validation should be conducted to ensure that the compounds are completely free of any toxic effects.

## 4. Conclusions

The phytochemical and biological investigation of the methanolic extract of *P. ecklonii* revealed that this plant is a rich source of abietane diterpenes with moderate alpha-glucosidase inhibitory activity as well as significant antioxidant activities. The present work is the first scientific report on the biological investigation of *P. ecklonii* constituents against alpha-glucosidase and alpha-amylase enzymes, and these results suggest that the methanolic extracts of this plant and/or its constituents might become potent natural therapeutic agents for oxidative stress. This study also linked the presence of the methoxyl group at 3.89 ppm on the benzoic acid to strong antioxidant and alpha-glucosidase activities. Therefore, compounds that demonstrated potent antioxidant activities may be very good candidates for controlling oxidative stress in diabetic patients.

## Figures and Tables

**Table 1:** Alpha-glucosidase and alpha-amylase inhibitory activities of *P. ecklonii* and its isolated compounds.

Items	Alpha-Glucosidase IC_50_ (µg/mL)	Alpha-Amylase IC_50_ (µg/mL)
**1**	NA	NA
**2**	NA	NA
**3**	41.3 ± 1.2	NA
**PE**	145.4 ± 3.1	NA
**Acarbose**	610.4 ± 1.0	10.2 ± 0.6

NA, not active at the test concentrations. The results are expressed as mean ± SEM for *n* = 3.

**Table 2:** Antioxidant activities of the isolated compounds.

Items	ORAC (µM TE/g)	TEAC (µM TE/g)	FRAP (µM AAE/g)
**1**	3942.9 ± 7.1	470.3 ± 2.2	1043.9 ± 2.6
**2**	1726.1 ± 2.6	1069.3 ± 2.4	1155.5 ± 2.3
**3**	28,726.1 ± 8.1	3526.1 ± 0.6	1455.4 ± 2.0
**EGCG**	3976.82 ± 3.8	4146.4 ± 19.8	7524.1 ± 4.9

**Table 3:** Predicted physicochemical and pharmacokinetic properties of the three isolated compounds and acarbose.

	Parvifloron D	Parvifloron F	Parvifloron G	Acarbose
MW	420.5	436.5	450.52	646.61
Fraction Csp3	0.35	0.35	0.37	0.92
#Rotatable bonds	4	4	5	9
#H-bond acceptors	5	6	6	18
#H-bond donors	3	4	3	14
TPSA	86.99	107.22	96.22	325.75
Consensus Log P	4.95	4.55	4.98	−7.16
ESOL Solubility Class	Poorly soluble	Poorly soluble	Poorly soluble	Highly soluble
GI absorption	High	High	High	Low
CYP2C9 inhibitor	Yes	Yes	Yes	No
Bioavailability Score (A value of 1 suggest optimal bioavailability)	0.55	0.55	0.55	0.17
PAINS #alerts	1 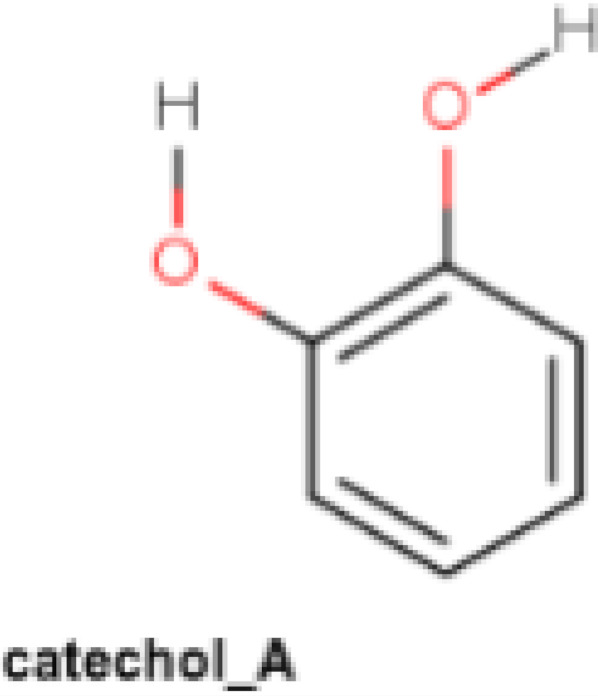	1 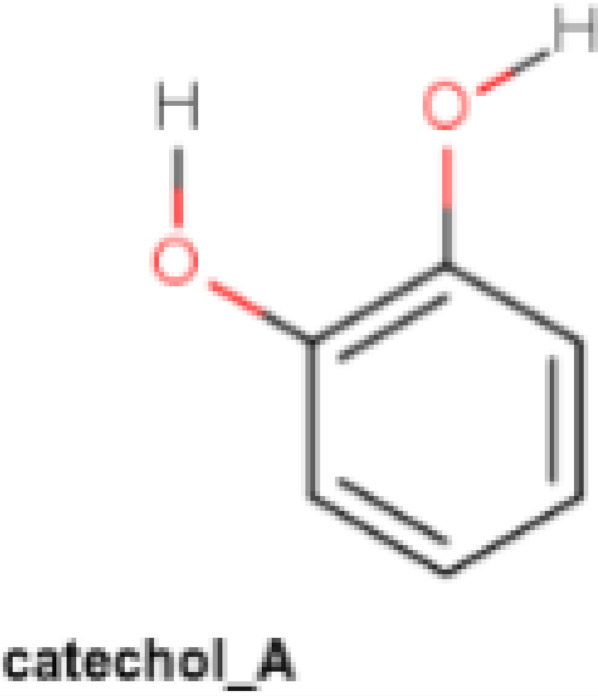	1 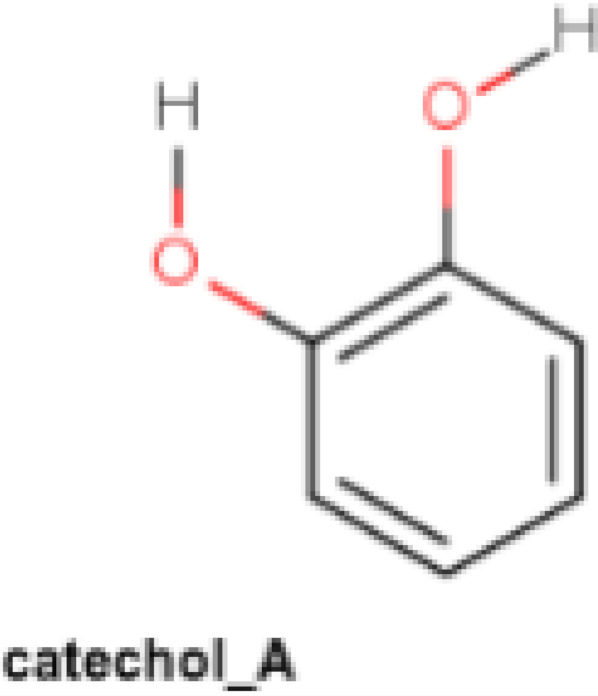	0
Mutagenicity/Tumorigenicity	High	High	High	None

## Data Availability

Data is contained within the article.
